# Upregulation of the B7/CD28 family member B7-H3 in bladder cancer

**DOI:** 10.3892/ol.2014.2828

**Published:** 2014-12-23

**Authors:** DEYAO WU, ZICHUN ZHANG, HUIXING PAN, YUANFENG FAN, PING QU, JIAN ZHOU

**Affiliations:** Department of Urology, The Fourth Affiliated Hospital of Nantong Medical College, Yancheng City No. 1 People’s Hospital, Yancheng, Jiangsu 224001, P.R. China

**Keywords:** bladder cancer, B7-H3, B7/CD28 family

## Abstract

Dysregulation of B7-H3 has been observed in a variety of types of human cancers. In the present study, the mRNA expression level of B7-H3 was analyzed in bladder cancer by performing semi-quantitative reverse transcription-polymerase chain reaction on clinical specimens from transitional cell carcinomas (TCCs) and their normal adjacent tissues (NATs). Immunohistochemical analysis was performed to compare the protein expression level of B7-H3 in TCCs and the paired NATs. The present study indicated that the B7-H3 mRNA expression level was significantly higher in the TCC samples compared with the paired NAT samples. Furthermore, immunohistochemical analyses indicated that the B7-H3 protein expression level was significantly upregulated in the TCC samples compared with in the paired NAT samples, indicating that B7-H3 dysregulation may be important in the progression of bladder cancer.

## Introduction

Bladder cancer is one of the most common malignancies worldwide. In males, bladder cancer is the fourth and seventh most common malignancy in the United States and the Western world, respectively, behind lung, prostate, colon, stomach, liver and esophageal cancers. Furthermore, bladder cancer is the second most common cause of mortality among genitourinary tumors, with ~72,570 new cases and 15,210 deaths occurring in males and females in 2013 (1–3). Transitional cell carcinoma (TCC) accounts for ~90% of cases of bladder cancer, whereas squamous cell carcinoma accounts for ~5% and adenocarcinomas ~1–2% of cases (4). At presentation, ~70% of cases of bladder cancer are non-muscle invasive (stages Tis, Ta and T1) which is typically low grade, multifocal, superficial and papillary, and 30% are muscle invasive (stages T2, T3 and T4), according to the TNM staging system (3). Numerous studies investigating the etiology of bladder cancer have been conducted; however, current understanding of the cellular and molecular mechanisms that underlie the processes of bladder cancer carcinogenesis and progression is poor.

The most common treatment for bladder cancer patients is endoscopic resection (5,6). Patients exhibiting non-invasive, low-grade bladder cancer typically undergo a treatment strategy of simple resection and fulguration of the tumor, followed by selective use of intravesical chemotherapy (7). Patients exhibiting the more aggressive muscle-invasive form of bladder cancer are usually treated with radical cystectomy of the primary tumor. However, despite this radical surgery, half of invasive bladder cancer patients develop subsequent metastatic disease (8). Therefore, bladder cancer patients require monitoring for cancer recurrence or progression. Furthermore, it is important to predict the prognosis of bladder cancer patients to enable the implementation of the most effective treatment strategy for prolonged survival.

Co-stimulation and -inhibition of T-cells is primarily generated by interactions between B7 immune regulatory ligands and cluster of differentiation (CD)28 receptors (9). Identification of new members of the B7/CD28 families have expanded the B7 family to include: B7h (CD275), B7× (B7-H4 or B7S1), B7-H3 (CD276), B7-1 (CD80), B7-2 (CD86), PD-L1 (CD274) and PD-L2 (CD273) (10). Phylogenetically, the B7 family is divided into three groups (11): Group I (B7-1, B7-2, and B7h) is involved in low-stage T-cell responses, as well as T- and B-cell interactions in lymphoid tissues (12); group II (PD-L1 and PD-L2) is involved in peripheral immune tolerance and T-cell impairment during chronic viral infections (13,14); and group III [B7-H3 (B7RP-2) and B7× (B7-H4, B7S1)], which contains the most recently identified members of the B7 family, is considered to attenuate peripheral immune responses via co-inhibition (9). The counter-receptors and precise roles of B7 proteins in T-cell regulation are yet to be defined; however, it is proposed that they are significant factors in the interaction between tumors and the immune system.

To the best of our knowledge, B7-H3 expression in bladder cancer has not yet been examined. Thus, the present study examines the mRNA and protein expression levels of B7-H3 in bladder cancer by performing semi-quantitative reverse transcription-polymerase chain reaction (RT-PCR) and immunohistochemical analysis, respectively, of clinical specimens from TCC samples and their normal adjacent tissues (NATs). The mRNA and protein expression levels of B7-H3 were significantly upregulated in the TCC samples compared with the paired NAT samples.

## Materials and methods

### Patients and clinical specimens

Seventeen tissue specimens were collected from bladder cancer patients of the Yancheng City No. 1 People’s Hospital (Yancheng, China). Following receipt of written informed consent, tissues were obtained under a general tissue collection protocol approved by the Institutional Review Board of Yancheng City No. 1 People’s Hospital. Tissues were immediately snap-frozen in liquid nitrogen.

### RNA extraction and complementary (c)DNA synthesis

Total RNA was extracted from the tissue samples using TRIzol reagent (Invitrogen Life Technologies, Carlsbad, CA, USA) and treated with RNase-free DNase (Sangon Biotech Co., Ltd., Shanghai, China) to remove genomic DNA contamination, according to the manufacturer’s instructions. The integrity and yield of RNA was quantified spectrophotometrically (UV-1601; Shimadzu Corporation, Kyoto, Japan) by measuring the absorbance at 260 nm (A260) and 280 nm (A280) to determine the A260/A280 ratio of pure RNA as ~1.8. RNA was stored at −80°C for subsequent analysis and cDNA synthesis was performed using the ReverTra Ace qPCR RT Kit (Toyobo Biotech Co., Ltd., Shanghai, China) in an Eppendorf RealPlex4S Mastercycler^®^ (Eppendorf, Hamburg, Germany).

### Semi-quantitative RT-PCR

Total RNA was isolated and analyzed for B7-H3 expression by semi-quantitative RT-PCR. The primer sequences for PCR were as follows: Forward, 5′-CAGGGCAGCCTATGACATTCCC-3′ and reverse, 5′-GTGACCAGCACATGCTTCCGTG-3′ for B7-H3; and forward, 5′-ATTCAACGGCACAGTCAAGG-3′ and reverse, 5′-GCAGAAGGGGCGGAGATGA-3′ for the GAPDH internal control. The following PCR cycling parameters were employed: 95°C for 5 min, followed by 42 cycles of 95°C for 45 sec, 56°C for 1 min and 72°C for 1 min, and then 72°C for 7 min. Equal volumes of the PCR reaction were subjected to electrophoresis on 2% agarose gels and PCR fragments were visualized by ethidium bromide staining. Images were captured on a gel documentation system and analyzed using an AlphaImager^®^ 2200 (Alpha Innotech Co., San Leandro, CA, USA).

### Immunohistochemical staining

At room temperature, the tissue samples were fixed in 4% 3-heptanone in phosphate buffered saline (PBS) for 24 h, embedded in paraffin, cut into sections (width, 5 μm) and mounted onto poly-L-lysine-coated slides. Initially, the slides were heated to 56°C in an oven for 2 h. Following dewaxing in xylene and rehydration in gradient alcohols, the slides were heated to boiling in 10 mmol/l sodium citrate buffer (pH 6.8) for 10 min using a microwave. Following inactivation of endogenous peroxidase activity with 3% hydrogen peroxide in methanol treatment, the samples were blocked using blocking solution (5% goat serum in PBS) for 20 min and incubated with primary mouse anti-human monoclonal B7-H3 antibodies (1:1,000; cat. no. BC062581; Bioworld Technology, Inc., St. Louis Park, MN, USA) overnight at 4°C. Following incubation, secondary biotinylated goat anti-mouse antibodies (1:1,000; cat. no. Bs10004; Bioworld Technology, Inc.) and ABC reaction solution (Beyotime Institute of Biotechnology, Shanghai, China) were applied sequentially and according to the manufacturer’s instructions. The slides were counterstained with hematoxylin, briefly washed with 30 mmol/l ammonium hydroxide and mounted with permanent mounting medium. Each experiment was performed in triplicate and conducted under identical conditions.

To quantify the immunostaining intensity, the integrated optical density (IOD) was calculated. Digitally fixed images were analyzed at a magnification of ×400 using an AxioImager A1 light microscope (Carl Zeiss AG, Oberkochen, Germany) equipped with Image-Pro Plus 6.0 software (Media Cybernetics, Rockville, MD, USA). For each sample, the IOD was calculated for the same sized arbitrary area. All data are presented as a mean value and statistical analysis was conducted to compare the results of the various experimental groups.

### Statistical methods

Data are presented as the mean ± standard deviation, and compared using a Student’s t-test in Stata software (version 8.2; StataCorp LP, College Station, TX, USA). P*<*0.05 was considered to indicate a statistically significant difference.

## Results

### mRNA expression level of B7-H3 in TCC and NAT samples

**Figure 1:**
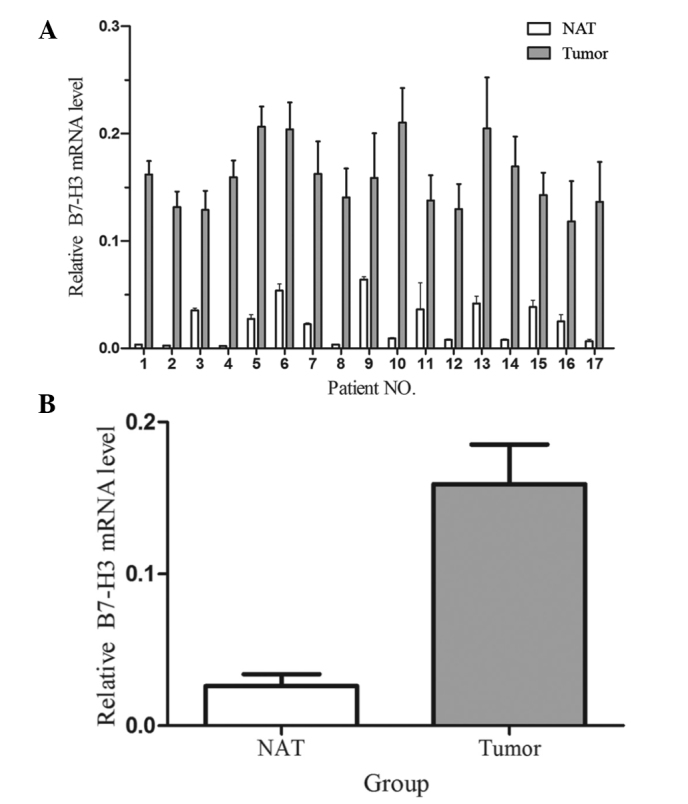
(A) Relative B7-H3 mRNA expression levels of 17 individual paired TCC and NAT samples. The band intensity signal in pixels for B7-H3 was divided by the respective intensity signal of GADPH. The relative B7-H3 mRNA levels were significantly higher in TCC samples compared with the paired NAT samples (P<0.05). (B) The mean relative B7-H3 mRNA level of the TCC samples compared with the NAT samples (P<0.05). TCC, transitional cell carcinomas; NAT, normal adjacent tissue.

The relative mRNA expression levels of B7-H3 in 17 TCC and the paired NAT samples were examined using semi-quantitative RT-PCR. In individual patients (Fig. 1A) and as a mean value (Fig. 1B), B7-H3 mRNA expression levels were significantly higher in TCC samples compared with the paired NAT samples (P<0.05).

### Protein expression level of B7-H3 in TCC and NAT samples

**Figure 2:**
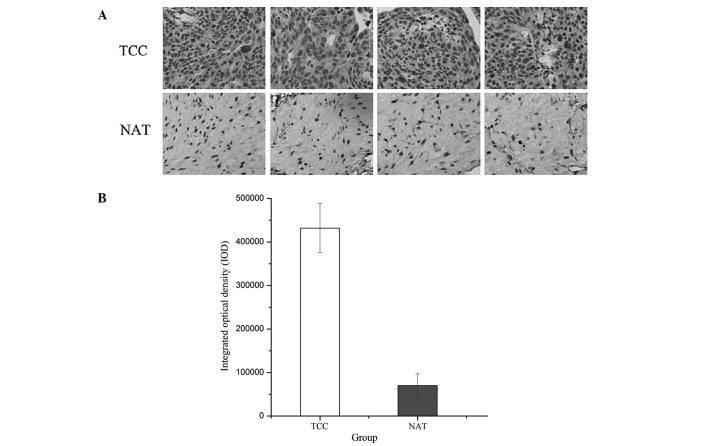
(A) Representative immunohistochemical staining for B7-H3 in TCCs and NATs. B7-H3 protein levels were significantly upregulated in the TCC samples compared with the paired NAT samples. (B) Integrated optical density represents the immunostaining intensity of B7-H3 in TCC and NAT samples. The mean B7-H3 protein level was significantly higher in the TCC samples compared with the paired NAT samples (P<0.05; staining, hematoxylin and eosin). Error bars present the standard error of the mean. TCC, transitional cell carcinomas; NAT, normal adjacent tissue.

The protein level of B7-H3 in TCCs and NATs was compared using immunohistochemistry (Fig. 2A). For quantitative analysis of immunostaining intensity, the IOD was calculated using Image-Pro Plus 6.0 software (Media Cybernetics; Fig. 2B). The results indicated that the B7-H3 protein level was significantly upregulated in the TCC samples compared with the paired NAT samples. Therefore, dysregulation of B7-H3 in TCCs may be important during the progression of bladder cancer.

### Association between B7-H3 mRNA expression level, and the tumor stage and grade of TCC patients

**Figure 3:**
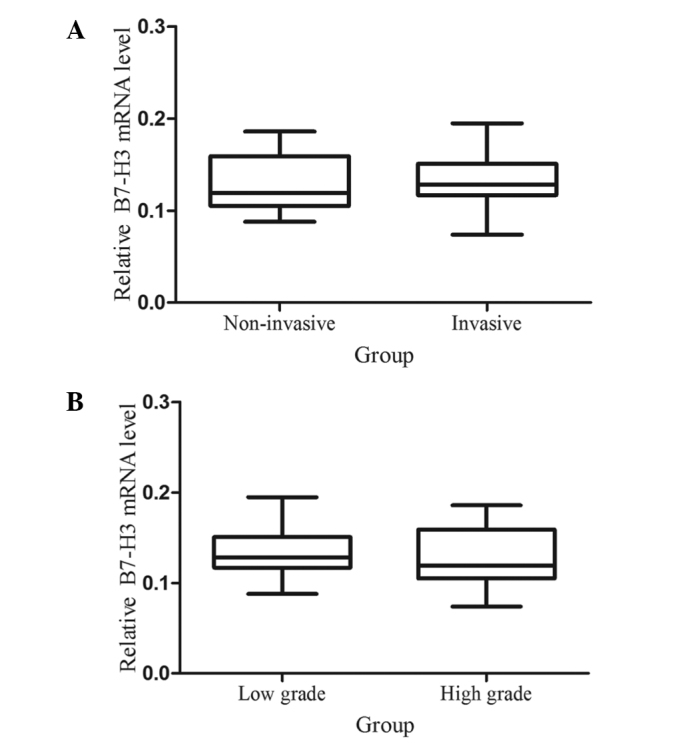
(A) Association between B7-H3 mRNA expression levels and tumor stage in bladder cancer patients. The relative B7-H3 mRNA expression level of patients exhibited no significant correlation with tumor stage (P>0.05). (B) Association between B7-H3 mRNA expression level and the tumor grade in bladder cancer patients. The relative B7-H3 mRNA expression level exhibited no significant correlation with tumor grade (P>0.05).

Previous studies have demonstrated that the expression of B7-H3 is associated with aggressive behavior in prostate cancer and clear cell renal cell carcinoma (15,16). Therefore, the present study investigated whether the mRNA expression level of B7-H3 is associated with the tumor stage and grade in bladder cancer patients. Statistical analysis indicated no significant association between the B7-H3 mRNA expression level, and the tumor stage (Fig. 3A) and grade (Fig. 3B).

## Discussion

In the present study, the mRNA and protein expression levels of B7-H3 were evaluated in TCC samples. In a significant fraction of bladder cancer patients, the mRNA and protein expression levels of B7-H3 were upregulated. To the best of our knowledge, this is the first report demonstrating alterations in the expression of B7-H3 in TCC.

Human B7-H3 is a member of the B7 family and shares 20–27% amino acid identity with other B7 proteins (17). B7-H3 exists in two forms which appear to be functionally identical; mouse B7-H3 contains extracellular IgV-IgC domains, whereas tandemly duplicated (IgV-IgC)_n_ domains are present in human B7-H3 due to exon duplication (18). B7-H3 was initially detected in interferon-γ (IFN-γ)-treated dendritic cells, and has since been identified to be inducible in monocytes, T cells, B cells and natural killer cells (19). Detailed analysis of the expression of B7-H3 in various tissues has revealed its immunobiological significance. At the transcriptional level, B7-H3 is expressed in the majority of organs (17,20), however, at the protein level, B7-H3 is expressed in human breast, bladder, liver, lung, lymphoid organs, placenta, prostate and testis (10). Furthermore, B7-H3 has been observed to be upregulated in prostate cancer (15), non-small cell lung cancer (21), gastric (22) and ovarian cancer (23). However, the specific cell type of B7-H3-positive cells in each of these organs has yet to be determined. The differential mRNA and protein expression patterns indicate that B7-H3 is post-transcriptionally regulated, although, the molecular mechanisms regulating B7-H3 expression remain unclear.

At present, there is no consensus regarding the physiologic or pathophysiologic roles of B7-H3, as immune stimulatory and inhibitory effects have been described for this ligand (24). The study that initially identified human B7-H3 demonstrated that it exhibits a co-stimulatory effect on T-cells (20) and a further *in vitro* study demonstrated that B7-H3 increased the proliferation of CD4 and CD8 T-cell populations and selectively stimulated IFN-γ production (25). In addition, B7-H3′s co-stimulatory effect on T-cell function is evidenced by reduced rates of acute and chronic cardiac allograft rejection in B7-H3 knockout mice (26). Furthermore, upon transient transfection of B7-H3 into melanoma cells, the induction of human primary CD8 cytotoxic T-cells was enhanced. Subsequently, mouse cancer models demonstrated that ectopic expression of B7-H3 results in the activation of tumor-specific cytotoxic T-cells that aid in slowing tumor growth or, in certain cases, completely eradicating the tumor. Furthermore, mice implanted with a B7-H3-transfected colon cell line exhibited significantly prolonged survival when compared with controls (27,28).

By contrast, various studies demonstrated B7-H3 acting as a T-cell co-inhibitor. In the majority of studies conducted thus far, human and mouse B7-H3 inhibit CD4 T-cell activation, and reduce the production of effector cytokines, such as IFN-γ and interleukin-4 (19,30,31). Inhibition of CD4 T-cell activation may be controlled via the N-[4-(5-nitro-2-furyl)-2-thiazolyl]acetamide, NF-KB and AP-1 signaling pathways, which T-cell receptors utilize to regulate gene transcription (10). In an independent study, the B7-H3 protein was upregulated in human malignant tumor cells, indicating an associated with increased disease severity (12). Prostate cancer patients exhibiting B7-H3 overexpression were at an increased risk of clinical cancer recurrence, metastatic development prior to surgery and cancer-related mortality. Furthermore, B7-H3 was upregulated in ovarian tumor vessels, which are associated with poor clinical outcome (15). These findings indicate that tumors may exploit B7-H3 as an immune evasion pathway via the downregulation of T-cell-mediated antitumor immunity. Thus, it was proposed that tumor-associated B7-H3 could be utilized as a novel therapeutic strategy as a targeted agent or for the enhancement of antitumor immunity (12). In the present study, the expression levels of B7-H3 were upregulated in bladder cancer, indicating that B7-H3 may act as a co-inhibitor in TCC. Although the present study identified that B7-H3 expression levels were higher in invasive and high grade TCCs, compared with non-invasive and low grade TCCs, the association of B7-H3 expression with these clinicopathological features was not statistically significant. The small sample size (n=17) may have contributed to the lack of significant results. Thus, further studies with larger sample sizes are required to clarify the present data.

In addition, further investigations are required to determine an arbitrary cutoff value for B7-H3 expression levels in the prognosis of bladder, to improve its clinical significance. In addition, further investigation may explain the mechanisms of B7-H3 upregulation and reveal a novel predictor of human bladder cancer.

In conclusion, our data demonstrate that B7-H3 is significantly upregulated in TCC samples compared with the paired NAT samples, indicating that higher expression of B7-H3 may be important during the progression of bladder cancer.
